# Is the Pain Stages of Change Questionnaire (PSOCQ) a useful tool for predicting participation in a self-management programme? Further evidence of validity, on a sample of UK pain clinic patients

**DOI:** 10.1186/1471-2474-7-101

**Published:** 2006-12-14

**Authors:** Jane L Carr, Jennifer A Klaber Moffett, Donald M Sharp, Derek R Haines

**Affiliations:** 1 Institute of Rehabilitation, University of Hull, UK; 2 Hull And East Yorkshire Hospitals NHS Trust, UK; 3 Centre for Pain Medicine, Hull And East Yorkshire Hospitals NHS Trust, UK

## Abstract

**Background:**

In the context of finite health resources, encouraging self-management of chronic conditions is important. Indeed, it is a key priority in the UK. An increasing number of self-management programmes are becoming available. However, patients may not always choose to participate in them. Some will prefer a more directed or medically orientated treatment. The acceptability of self-management programmes for patients suffering from chronic pain is an important issue. Few measures exist that examine the process of change to a self-management approach. The Pain Stages of Change Questionnaire (PSOCQ) was evaluated for this purpose in the present study. Hypotheses were centred around criterion and construct validity of the PSOCQ.

**Methods:**

A sample of pain patients was surveyed about their interest in participating in a lay-led self-management programme ('the Expert Patients Programme'). In addition, participants completed two psychometric measures: the Pain Stages of Change Questionnaire (PSOCQ) together with the Chronic Pain Acceptance Questionnaire (CPAQ). This is the first study as far as we are aware to examine these two scales together. The psychometric properties of the PSOCQ were examined. Analyses focused on the associations between the PSOCQ scores and interest in participating in the self-management programme. Further associations were examined between the PSOCQ and the Chronic Pain Acceptance Questionnaire.

**Results:**

The results demonstrated qualified support for the PSOCQ, in particular the Contemplation sub-scale. There was a significant positive association between interest and likelihood of joining the self-management programme and contemplation scores. The action and maintenance sub-scales appeared to be measuring a unitary dimension. The associations between the PSOCQ and the Chronic Pain Acceptance Questionnaire were in the directions predicted. The limitations of the study were discussed.

**Conclusion:**

The results showed some support for the PSOCQ as a potentially useful tool in assessing who may or may not be likely to join a self-management course.

## Background

Self-management is increasingly emphasised as a key component in chronic disease treatment, including chronic pain management. Most psychological programmes designed to help patients alter behavioural and perceptual patterns surrounding pain also aim to foster self-reliance and reduce medical intervention. The effectiveness of cognitive behavioural programmes where self-management skills training is incorporated is well documented [1,2]. CBT programmes are not available for all chronic pain sufferers but health professionals dealing with chronic pain patients are encouraged to promote self-management in a number of different ways [3-5].

It is inevitable that some patients will adapt well to this approach while others will be less able to do so. Patients may be included in a self-management programme before they are ready and able to cope with it and it may thus be unhelpful both for the patients and from a resource management perspective. It is difficult to know who may or may not respond and there are few measures in existence that examine patients' orientation towards a self-management approach. Typically psychometric measures tend to be outcome focused rather than process focused.

One potential measure that has been developed is the Pain Stages of Change Questionnaire [6]. This is based on the transtheoretical model of behaviour change [7] in which individuals are seen to progress through a number of stages involving decisions about change. The first stage *precontemplation *is a stage in which little action is being considered. The second stage *contemplation *is one in which there is an intention to change and the third and fourth stages (*action *and *maintenance*) are involved with achieving and maintaining change. The Pain Stages of Change Questionnaire is likewise based on similar stages of change (pre-contemplation, contemplation, action and maintenance) for use in pain self-management [6,8,9]. The intention was to provide a tool for clinicians to identify patients' current stage in terms of readiness to change to a self-management approach to pain. Previous studies have established some reliability and validity although there appears to be some doubt as to the number of appropriate factors in the PSOCQ [10]. There is also some doubt as to whether it is possible to classify patients according to a distinct stage of change [9,11].

What has been proposed as a possible precursor of change to a self-management approach is acceptance of some level of pain [12]. Several studies have recently focused on examining the important effects of acceptance and non-acceptance of pain [13-15]. Emphasis on finding a cure or on controlling pain appears to be associated with greater pain and disability [16] whereas acceptance has been associated with reduced pain and disability, better adjustment and better work status [13,14,16]. The Chronic Pain Acceptance Questionnaire (CPAQ) based on two constructs, namely activity engagement and pain willingness, has been developed to measure acceptance of chronic pain [12]. This is a relatively new scale but some reliability and validity has been demonstrated. As a construct it has been found to be distinctly separate from coping [13].

Intuitively if adaptation to pain is seen to be largely a product of acceptance and change then there should be an association between scores on pain acceptance and scores on readiness to change. This is the first study as far as we are aware to examine the relationship between these two concepts based on the Pain Stages of Change Questionnaire and the Chronic Pain Acceptance Questionnaire.

The following hypotheses were tested: (i) Scores on the *pre-contemplation *sub-scale would be negatively associated with interest in a self-management programme; (ii) Scores on the *contemplation *sub-scale would be positively associated with interest in participating in a self-management programme; (iii) Patients scoring high on *action *and *maintenance *subscales would be oriented towards joining the self-help programme; (iv) Significant associations between acceptance and change would be found. Specifically, that scores on pain acceptance would be negatively associated with scores on the *pre-contemplatio*n sub-scale and positively correlated with scores on the *contemplation*, *action *and *maintenance *sub-scales.

## Methods

### Participants

All patients registered at a Pain clinic during a 4-week period (including new attendees and follow-up patients) were invited to participate in the study. The majority were suffering from chronic non-malignant pain, mostly involving the musculoskeletal system. Most interventions at the clinic were pharmacological in nature.

### Procedure

Patients received a leaflet about a self-management programme with a covering letter, together with a number of questionnaires. The aim was to ascertain their views about self-management and the likelihood of their attending the course. The self-management course was not part of the pain clinic's psychological or educational based programmes. It was a generic course in its pilot stage developed for the benefit of people with chronic conditions called the Expert Patients Programme (EPP). This is a nationally based self-management training initiative based on the chronic disease self-management programme (CDSMP) developed originally at Stanford University USA [17]. The programme is lay-led and involves six sessions of two and half hours each. It is based on a well-tested model that has been used in the United States for a number of years [17,18]. It was recently adopted by the UK Department of Health in order to help lessen the impact of chronic disease [19]. Ethical approval for this study was provided by the South Humber Local Research Ethics Committee (reference number 04/Q1105/27). Informed consent was not required by the Ethics Committee in this study.

### Self-Management Programme leaflet

This was the generic leaflet for the national pilot programme of the self-management programme, (known nationally as the *Expert Patients' Programme*). In this leaflet the programme was described as "a course where you take control of your condition and make a difference to your life". It provides a list of what the course offers, e.g.: "manage your symptoms, deal with stress, depression and low self-image, manage pain, develop coping skills, relax, eat healthily, work more closely with those caring for you, plan for the future".

### Measures

#### Survey of interest in the self-management programme

All patients were asked to respond to a survey asking for their views on the self-management programme. It was designed to elicit opinions about how interested an individual was in the programme and whether he or she might join a class. All questions were on a 5-point likert type scale and included "how interested are you in courses of this nature", "How helpful do you think this course might be for you?" "How comfortable are you with the idea of attending a course?" (each with a scale ranging from "a lot" to "not at all"), "How likely are you to join this course?" (with a scale ranging from "very likely" to "very unlikely"), and "when might you consider joining this course" (with a scale ranging from "immediately" to "never").

#### Readiness to change

The Pain Stages of Change Coping Questionnaire (PSOCQ) was the measure chosen to examine patients' readiness to adopt a self-management approach to chronic pain. It was developed by Kerns et al [6] and is based on the transtheoretical model of behaviour change and stages theory [7]. The PSOCQ has been developed to incorporate 4 dimensions each representing 4 (distinct) stages: *pre-contemplation*; representing a stage whereby the pain sufferer is not considering changing behaviour, *contemplation*; whereby changes in behaviour are being considered (albeit with an inclination to favour medical intervention), *action*; whereby active steps are being taken to change behaviour, and *maintenance*: whereby attempts to maintain those changes are evident. The questionnaire was based on a 5-point likert type scale from 'strongly disagree' (1) to 'strongly agree' (5). The PSOCQ was examined for the ability to classify patients into a distinct stage. In accordance with Jensen et al [9] patients were classified according to their highest PSOCQ score.

#### Pain Acceptance

The Chronic Pain Acceptance Questionnaire (CPAQ revised method) was used to measure patients' acceptance of pain [12]. This is a 20-item measure of acceptance of pain. It has two sub-scales representing *activity engagement *and *pain willingness*. Questions are based on a 7- point scale from 'never true' (0) to 'always true' (6). Acceptance of chronic pain has been shown to be negatively associated with avoidance of pain and positively associated with better mental well being, fewer visits for professional help, and fewer analgesics [20]. It has also been shown to discriminate between those who are functioning well and those who are not [20]. Psychometric work on the CPAQ is relatively limited but the scales are shown to be internally consistent [15].

## Results

### Patient characteristics

Of the 320 or more patients who were sent a leaflet about the self-management programme and questionnaire only 98 were returned (31%), of which 96 were complete. The majority of respondents were female (68%). The mean age was 53 (range 21 to 82). More than a third of the sample had chronic pain lasting more than 10 years, with 34% reporting between 2–5 years, 26% reporting 5–10 years with only 4% having had the pain for 1–2 years and 2% having had the pain for less than a year. A majority had been registered at the Centre for Pain Medicine for more than a year (65%). Approximately 17% had been registered for between 6 months and 1 year and around 18% had been registered for less than 6 months, (approximately 3% were newly registered patients). Participants were recipients of largely passive interventions for pain control.

Of those who responded to questions on educational attainment, a large proportion (66%) had gained some educational certificates, 60% of whom had professional or vocational qualifications and around 10% had a degree or the equivalent of a degree. Approximately a third of respondents did not have any educational certificates.

### Associations among the PSOCQ scales

Table 1 shows the results of the correlational analysis using Pearsons Correlation coefficients, between the PSOCQ subscales. Unacceptably high correlations were found between the action and maintenance subscales (r = 0.75) suggesting that these two scales were measuring a unitary dimension.

### Principal components analysis

Principal components analysis, with varimax rotation produced a 7 factor solution using the eigenvalues >1 criteria. This explained 65% of the variance, although a 3-factor solution seemed more appropriate following examination of the scree plot and in view of the small number of items loading onto the other factors. Approximately 47% of the variance was explained by the 3-factor solution. The factor structure shown in Table 2 provides support for the factorial validity of the PSOCQ subscales '*precontemplation*' and '*contemplation*' subscales. All items from the contemplation subscale, except item 9 ('I have been thinking that doctors can only help so much in managing my pain...') loaded onto factor 1 and accounted for 22% of the variance. Factor 3, the *precontemplation *subscale had loadings of 0.5 or greater for all of the items listed on this dimension, except item 3 ('everybody I speak with tells me I have to learn to live with my pain....'). This item loaded independently onto factor 7. The items representing the 'action' and 'maintenance' subscales each loaded onto factor 2 and accounted for 18% of the variance. Only 1 item from these two subscales had a loading of less than 0.35 ('I am learning to help myself control my pain without doctors'), which loaded onto factor 5.

Overall there was considerable support for the structure of the PSOSQ with the exception of the 2 separate subscales of 'action' and 'maintenance', which appeared, in this sample, to be measuring a unitary dimension.

### Associations of the PSOCQ scales with interest and likely up-take of the self-management programme

There was a significant positive association between expressed interest and likelihood of joining the self-management programme and contemplation scores as shown in Table 3. High scorers on this sub-scale were more likely to state they were interested in the self-management programme and would join a class. There were no significant associations between interest and likelihood of joining the self-management course and other PSOCQ scores.

### Differences in PSOCQ scores between those interested and those uninterested in the self-management programme

Pain patients who expressed a likely intent to join an EPP class scored significantly higher on the contemplation scale (mean = 37.26) compared with those who expressed uncertainty or said they were unlikely to join (mean = 31.78), (CI = 2.51 to 8.45). No significant differences were found for the other sub-scales.

### Classification of patients into one stage

Most patients were in the *contemplation *stage (forty three per cent), thirty seven per cent were in the *action/maintenance *stage and twenty per cent were in the *pre-contemplation *stage. There was some support for the classification of patients in this way. Table 4 shows the results of the analyses comparing the PSOCQ scores in the classified groups.

### Relationship of PSOCQ with Chronic Pain Acceptance Questionnaire (CPAQ)

Table 5 shows the relationship of the PSOCQ with the CPAQ. As expected there was a significant negative correlation between *pre-contemplation *and pain acceptance and a similar but weaker association between *contemplation *and pain acceptance. *Action/maintenance *was positively associated with pain acceptance. *Pre-contemplation *was negatively associated with pain willingness and, to a lesser degree but in the same direction, with activities engagement, *contemplation *was also significantly negatively associated with pain willingness (to a lesser degree) but not with activities engagement. *Action/maintenance *was positively associated with activities engagement but not with pain willingness.

## Discussion

The results of this study provide some support for the PSOCQ in a UK sample. The contemplation scale may have particular value in predicting who may or may not be ready to participate in a self-management programme. This finding is consistent with previous studies [21,22]. Further support for the contemplation sub-scale was demonstrated by sound psychometric properties, in line with previous studies [9,10,23].

Psychometric support for the *pre-contemplation *subscale was also found. Unlike previous studies however this sub-scale failed to predict level of interest in the self-management class. There may be several reasons for this. A relatively low proportion of patients were classified as being in this stage. In addition, it could have reflected some ambivalence (for some patients) about how the course was going to help them. Lay-led self-management courses are a fairly novel trend in the UK and patients only had the leaflet for reference purposes.

The unacceptably high correlations observed between action and maintenance scales were in line with previous findings [9,10,24]. PCA also confirmed the unidimensional nature of these sub-scales. Scores on the *action *and *maintenance *scales did not show any pattern of association with expressed interest and likely up-take of the self-management programme. This is consistent with previous findings [22]. Action and maintenance combined probably represent the point at which individuals are already doing things for themselves. The fact that they are already engaged in self-help, may explain their ambivalence regarding the offer of additional advice on helpful strategies.

In general the associations found between the PSOCQ and the CPAQ were in the hypothesised direction. As expected, scores on pre-contemplation were negatively associated both with pain willingness and with engagement in activity. This is consistent with the idea that people in the pre-contemplation stage are seeking a medical cure, are less active and are less willing to accept some level of pain [6,9]. The negative correlation observed between contemplation scores and pain willingness possibly suggests that those who are less willing to accept pain are motivated to look at other ways of dealing with the problem. These findings are consistent with the hypothesised nature of the contemplation stage, which suggests that patients in this stage are possibly still looking for a 'cure'. The lack of association observed between the contemplation scale and activities engagement would be expected if this scale represents the point at which little action had as yet been taken. The positive association found between action/maintenance and activities engagement is consistent with the idea that people in this stage are more likely to be functioning despite the pain and not trapped in a cycle of pain and disability.

One of the problems raised about the PSOCQ is the scale's lack of a strong relationship with adaptive behaviours [9,23-25]. However, it might be better conceptualised as a measure representing cognitive shift in perspective, perhaps in terms of thinking about alternative ways of dealing with the pain problem, rather than a readiness to adopt specific coping behaviours or actions. This would have some practical value since it is difficult to determine what particular behaviours pain patients may prefer from a self-management perspective. The sub-scale may be valuable in identifying those patients who are more open to different ways of dealing with pain. In this study high scores on the contemplation sub-scale indicated a possible willingness to try something independent of medical management, that something else being a lay-led self-management programme.

Validation studies on the PSOCQ have often been based on samples demonstrating good to high levels of education [6,9,22]. This was probably because studies using the PSOCQ were dependent upon volunteers to a self-management ethos. This philosophy may in fact be more acceptable to the better-educated or more middle class patient [26]. In this present study respondents were a relatively well-educated pain clinic sample in a UK city characterised by large pockets of severe social deprivation. The sample also had a relatively high proportion of female participants. Future studies may need to address these validity issues.

There are several limitations to this study. The results are constrained by the sample size. Other studies have been equally constrained [10,22]. The response rate was low and in view of this it is difficult to know to what extent the sample is representative of the clinic population. This was a clinically opportunistic sample and it was not possible to follow-up non-respondents due to ethical approval constraints.

## Conclusion

Despite the limitations the results of this study found some support for the PSOCQ as a useful tool in assessing who may or may not be likely to join a self-management course. The associations found in relation to the Chronic Pain Acceptance Questionnaire were consistent with the hypothesised nature of readiness to change and add overall strength to the validity of the PSOCQ.

## Competing interests

The author(s) declare that they have no competing interests.

## Authors' contributions

JLC and JAKM conceived the study and participated in its design. JLC carried out the statistical analyses and wrote the first draft and successive drafts of the manuscript JLC, together with DMS and JAKM interpreted the findings and produced successive drafts. DRH participated in the design of the study. All authors read and approved the manuscript.

## Pre-publication history

The pre-publication history for this paper can be accessed here:



## Figures and Tables

**Table 1:** Pain Stages of Change Questionnaire inter-scale correlation coefficients (using Pearson Correlation Coefficients)

PSOCQ scale	Contemplation	Action	Maintenance
Precontemplation	0.10	-0.23*	-0.34**
Contemplation		0.27**	0.07
Action			0.75**

* p < 0.05

** p < 0.01

**Table 2:** Items and factors loadings of the PSOCQ scale (showing first 3 factors only)

	**Factor 1**	**Factor 2**	**Factor 3**
**Precontemplation**			
I have tried everything that people recommended...	0.23	-0.21	**0.55**
My pain is a medical problem and I should be....	-0.03	-0.13	**0.40**
Everyone I speak with tells me I have to learn....	-0.02	-0.17	**0.08**
I still think despite what doctors tell me there must...	-0.03	0.00	**0.83**
The best thing I can do is find a doctor who can ....	0.28	-0.10	**0.79**
Why can't someone just do something to take away...	0.31	0.03	**0.65**
All of this talk about how to cope better is a waste...	-0.46	-0.15	**0.05**
			
**Contemplation**			
I have been thinking that the way I cope with my pain...	**0.77**	0.28	0.15
I have recently realised there is no medical cure...	**0.65**	-0.15	-0.06
Even if my pain doesn't go away I am ready to....	**0.69**	0.11	0.00
I realise now that it is time for me to come up with...	**0.80**	-0.07	0.26
I am beginning to wonder if I need to get some help...	**0.84**	0.02	0.14
I have recently figured out that it's up to me to....	**0.41**	0.09	-0.07
I have recently come to the conclusion that its time...	**0.81**	0.09	0.05
I am starting to wonder whether it's up to me to ...	**0.49**	0.12	-0.04
I have been thinking that doctors can only help....	**0.16**	0.14	-0.18
I have been wondering if I there is something I...	**0.55**	-0.02	0.17
			
**Action**			
I am developing new ways to cope with my pain	0.09	**0.67**	-0.01
I have started to come up with some strategies	-0.13	**0.52**	-0.09
I am getting help learning some strategies for coping	0.17	**0.70**	-0.21
I am learning to help myself control my pain without...	0.02	**0.18**	-0.03
I am testing out some coping skills to manage my ....	0.35	**0.77**	-0.04
I am learning ways to control my pain other than...	-0.03	**0.58**	0.05
			
**Maintenance**			
I have learned some good ways to keep my pain...	-0.03	**0.56**	-0.16
When my pain flares up I find myself automatically....	0.01	**0.36**	-0.06
I am using some strategies that help me better deal.	-0.17	**0.62**	-0.19
I use what I have learned to help keep my pain ...	-0.05	**0.59**	-0.16
I am currently using some suggestions people have...	-0.22	**0.81**	0.00
I have incorporated strategies for dealing with...	-0.14	**0.65**	0.00
I have made a lot of progress in coping with my...	-0.28	**0.45**	-0.37

**Table 3:** Coefficients between CSOCQ and expressed interest and likelihood of joining the self-management programme

**PSOCQ scale**	**Expressed interest in EPP**	**Expressed intent to join**
Pre-contemplation	0.03	0.06
Contemplation	0.51**	0.55**
Action/maintenance	-0.06	0.06

** 0.01 level

**Table 4:** Comparisons between classified stages on PSOCQ

**Classified Stage**				
**Mean PSOCQ scale score**	Precontemplation	Contemplation	Action/Maintenance	F-value

Precontemplation	3.79^3^	2.94^2^	2.55^1^	27.90*
Contemplation	3.02^1^	4.01^2^	3.00^1^	34.55*
Action/maintenance	2.60^1^	2.84^1^	3.47^2^	12.81*

*p < 0.001

Means with different superscripts differ significantly using Tukey HSD test (p < 0.05)

**Table 5:** Correlation Coefficients between PSOCQ sub-scales and CPAQ sub-scales

**PSOCQ sub-scales**	**CPAQ**		
	Acceptance Total	Pain Willing	Activities engagement

Pre-contemplation	-0.47**	-0.51**	-0.26*
Contemplation	-0.24*	-0.41**	-0.03
Action/maintenance	0.29*	0.08	0.38*

* 0.05 level

** 0.01 level
